# Changes in the prevalence and profile of users of contraception in Britain 2000–2010: evidence from two National Surveys of Sexual Attitudes and Lifestyles

**DOI:** 10.1136/bmjsrh-2019-200474

**Published:** 2020-01-21

**Authors:** Rebecca S French, Lorna Gibson, Rebecca Geary, Anna Glasier, Kaye Wellings

**Affiliations:** 1 Faculty of Public Health, Environments and Society, London School of Hygiene & Tropical Medicine, London, UK; 2 Faculty of Epidemiology and Population Health, London School of Hygiene & Tropical Medicine, London, UK

**Keywords:** contraceptive methods, prevalence, trends, probability sample survey

## Abstract

**Aim:**

To describe prevalence and trends in contraceptive method use in Britain through a comparison of the second and third National Surveys of Sexual Attitudes and Lifestyles (Natsal-2 and Natsal-3).

**Methods:**

Cross-sectional probability sample surveys. General population sample of women aged 16–44 years, resident in Britain, with ever-experience of vaginal sex and, for analysis by sociodemographic characteristics, vaginal sex in the last year. Main outcome measure was current contraceptive method use (‘usual these days’), categorised by effectiveness.

**Results:**

Prevalence of current contraceptive use among women who had ever had vaginal sex declined between Natsal-2 and Natsal-3, 83.5% (95% CI 82.4 to 84.5) and 76.4% (95% CI 75.0 to 77.7), respectively. The condom and oral contraceptive pill remain the most commonly used methods. One in five women reported use of a most effective method. While no difference was found between surveys in use of most effective methods, a decline in sterilisation use was compensated by an increase in long-acting reversible contraceptive (LARC) use. Increased LARC use was particularly evident among under-25s compared with women aged 40–44 years (OR 11.35, 95% CI 3.23 to 39.87) and a decline was observed among those with two or more children relative to those with none (OR 0.21, 95% CI 0.13 to 0.35).

**Conclusions:**

Strategies to improve access to LARC methods have been particularly successful in increasing uptake among young people in the first decade of the 21st century. Whether this trajectory is maintained given changing sociodemographic characteristics and more recent financial cuts to sexual health service provision will warrant investigation.

**Key messages**

- The condom and the oral contraceptive pill continue to be the most commonly used contraceptive methods in Britain.
- A significant increase in long-acting reversible contraceptive (LARC) use has been observed, particularly among women under 25 years of age, suggesting strategies to increase uptake have been successful.
- A significant increase was also observed in the proportion of women reporting no current contraceptive use.

## Introduction

Unintended pregnancy continues to be a public health problem in Europe.^1^ In Britain, an estimated one in five pregnancies are not planned.^2 3^ While contraception can prevent unintended pregnancy, the effectiveness and continuation rates of available methods vary greatly. The last half century has seen major changes in patterns of fertility and its control. The interval between onset of sexual activity and childbearing has widened, and desired and actual family size has decreased, thus increasing the time period during which effective contraception is most required.^3^

The 21st century has seen a range of policy-related initiatives aimed at reducing rates of unintended pregnancy in Britain, many of them specifically focused on increasing awareness and uptake of long-acting reversible methods of contraception (LARC). Mounting evidence of the cost effectiveness of LARC methods prompted the National Institute for Health and Care Excellence (NICE) to publish guidance in 2005 recommending the increased use of such methods.^4^ The income of general practitioners was linked to performance against Quality and Outcome Framework (QOF) goals, and in 2009 a new set of indicators on contraception was introduced, including provision of information about LARC methods.^5 6^

Thus far, there have been few national data with which to assess progress towards the goals of these initiatives. Efforts to do so have relied on routinely collected data such as prescribing rates in primary care which provide information about rates of method use, but not users.^7^ Survey data on specialist contraceptive service use has reported an increase in LARC method use over the last decade.^8^ However, it offers limited opportunities to explore correlates of use associated with user sociodemographic characteristics and sexual and reproductive behaviour, and does not include women who obtain methods from other suppliers, such as general practice and pharmacies.

Data from the two most recent National Surveys on Sexual Attitudes and Lifestyles (Natsal-2 and Natsal-3) provide an opportunity to examine the mix of contraceptive methods used by women in Britain at the start of the 21st century and to describe changes in patterns of use. Specifically, the data enable us to determine whether there has been a change in the use of the most effective contraceptive methods, how this varies between subgroups of women, and with what apparent effect on use of other methods. In this article we describe trends in contraceptive method use in Britain between Natsal-2 and Natsal-3 with the aim of exploring how changes in patterns of fertility and in policy may have impacted on method use, in particular LARC methods.

## Methods

Natsal is a clustered and stratified probability survey of the sexual attitudes and behaviours of men and women resident in Britain. It has been carried out roughly decennially in 1990 (Natsal-1), 1999–2000 (Natsal-2) and 2010–2012 (Natsal-3). Data from Natsal-1 have not been included in this analysis since contraceptive implants were not available in Britain until 1991 and the levonorgestrel-releasing intrauterine system (LNG-IUS) was not marketed until 1995. In Natsal-2, 11 161 participants aged 16–44 years (6399 women and 4762 men) were interviewed; Natsal-3 included 15 162 participants aged 16–74 years (8869 women and 6293 men). Households within postcode sectors, the primary sampling units, were selected and one eligible adult at the address randomly selected. Data were weighted to take account of unequal probabilities of selection and non-response. Computer-assisted personal interviews (CAPI) were used for data collection and computer-assisted self-interviews (CASI) were used for more sensitive questions. The response rates for Natsal-2 and Natsal-3 were 65.4% and 57.7%, respectively, and in Natsal-3 the co-operation rate (ie, of all eligible addresses contacted) was 65.8%. Full details of the survey methods have been described elsewhere.^9^

In this article we use responses to the Natsal question about contraceptive methods currently used: “Which would you say is your most usual method these days?”. Participants who reported in CASI ever having had sexual intercourse with an opposite sex partner were presented with a list of different contraceptive methods to select. Analysis was first confined to women aged under 45 years who reported ever having had vaginal sex to provide population estimates on current use of different contraceptive methods and to investigate changes in use between Natsal-2 and Natsal-3. When examining user characteristics by contraceptive method use the analysis was then confined to women who had vaginal sex in the last year and those who were not pregnant at the time of interview. Although participants were asked in Natsal-3 whether they or their partner were trying to conceive and whether they had had a hysterectomy, these questions were not asked in Natsal-2. Therefore, women in Natsal-3 who reported that they were trying to conceive or had had a hysterectomy were kept in the analysis to allow comparison between the two surveys.

Contraceptive methods were characterised by level of effectiveness into: Most effective (female/male sterilisation, intrauterine device (IUD), intrauterine system (IUS), implant); Effective (oral contraceptive pill, injection, transdermal patch); Less effective (condom, Femidom, cap, spermicides, rhythm method, withdrawal) and No method used. The categorisation was based on typical rather than perfect user failure rates.^10^ In analysis, the categories were grouped hierarchically so that if more than one method was reported, the more effective method took precedence. The injectable contraceptive depot medroxyprogesterone acetate (DMPA, Depo-Provera) was not included in the most effective method category, despite being categorised as a LARC by NICE, since this method is generally placed in the same tier of effectiveness as oral contraceptive pills. Although the contraceptive effect of injections lasts 12 weeks, the method can be stopped without the need to see a health provider and discontinuation rates are similar to those of oral contraception. The use of DMPA, in contrast to other LARCs, has not been shown to reduce unintended pregnancy rates.^11^ When examining user characteristics, permanent methods (ie, female and male sterilisation) and LARC methods were analysed separately.

Usual contraceptive method use was examined by demographic variables: age, ethnicity, religion, academic qualifications, Index of Multiple Deprivation, and relationship status. We also examined sexual and reproductive health behaviour variables: sexually transmitted infection (STI) in the last year, frequency of sex in the last 4 weeks, and parity.

All statistical analyses were conducted using the Stata (version 13) (StataCorp, College Station, TX, USA) survey commands, in order to account for the weighting, clustering, and stratification of the survey data. Prevalence of method use was estimated. Bivariate analysis was used to describe associations between method use and selected sociodemographic characteristics and sexual and reproductive behaviours. The odds ratios (ORs) for the difference in prevalence between Natsal-2 and Natsal-3 were examined; 95% confidence intervals (95% CIs) are used throughout.

## Results

In Natsal-3, 5237 of the 5842 women aged 16–44 years reported that they had ever had vaginal intercourse. Over three-quarters of these women (76.4%, 95% CI 75.0 to 77.7) reported current use of any contraception. In Natsal-2, 5178 of the 6399 women reported that they had ever had vaginal intercourse. Current contraceptive use was reported by 83.5% (95% CI 82.4–84.5) of women, significantly higher than the prevalence observed in Natsal-3. The condom and oral contraceptive pill were the methods most commonly used by women and this was observed in Natsal-2 and Natsal-3 (table 1). In the most effective contraceptive method category, male sterilisation was replaced by implants as the most commonly cited method over the last decade (8.9% and 0.3%, respectively, in Natsal-2 compared with 4.9% and 5.4%, respectively, in Natsal-3). Use of the patch, female condom, cap or diaphragm and spermicides was very low in both surveys. Around one in five women reported in Natsal-3 that they had not used any method of contraception in the last year, however 7.4% (95% CI 6.6 to 8.3) of all women reported no vaginal sex in the last year, 7.6% (95% CI 6.8 to 8.5) were trying to conceive, 4.4% (95% CI 3.8 to 5.0) were pregnant at the time of interview and 1.4% (95% CI 1.0–2.0) had had a hysterectomy. Women were not asked in Natsal-2 whether they were trying to conceive or if they had had a hysterectomy.

**Table 1:** Usual use of contraceptive methods among women aged 16–44 years who ever reported vaginal intercourse: Natsal-2 and Natsal-3

Denominator (unweighted, weighted)*	Natsal-2: usual use		Natsal-3: usual use	
	5178, 4651		5237, 3657	
	n	% (95% CI)	n	% (95% CI)
Most effective methods	1141, 1077	21.5 (20.4 to 22.8)	944, 762	21.1 (19.7 to 22.6)
Intrauterine device	265. 221	4.4 (3.9 to 5.0)	230. 187	5.2 (4.4 to 6.1)
Hormonal intrauterine system (Mirena)	44, 35	0.7 (0.5 to 1.0)	105, 96	2.6 (2.1 to 3.3)
Implant	15, 14	0.3 (0.2 to 0.5)	348, 194	5.4 (4.8 to 6.0)
Male sterilisation	445, 446	8.9 (8.1 to 9.8)	162, 177	4.9 (4.1 to 5.8)
Female sterilisation	382, 370	7.4 (6.6 to 8.3)	106, 114	3.2 (2.6 to 3.9)
Effective methods†	1940, 1747	34.9 (33.5 to 36.4)	1953, 1194	33.1 (31.6 to 34.6)
Pill	1769, 1599	32.0 (30.6 to 33.4)	1741, 1064	29.5 (28.1 to 30.9)
Injections	171, 148	3.0 (2.5 to 3.5)	210, 128	3.6 (3.0 to 4.1)
Less effective methods†	1641, 1417	28.3 (27.0 to 29.7)	1356, 940	26.1 (24.6 to 27.5)
Male condom	1360, 1184	23.7 (22.4 to 25.0)	1229, 833	23.1 (21.7 to 24.5)
Cap/diaphragm†	45, 31	0.6 (0.4 to 0.9)	–	–
Natural family planning (rhythm)	104, 85	1.7 (1.3 to 2.1)	37, 35	1.0 (0.7 to 1.4)
Withdrawal	173, 150	3.0 (2.6 to 3.5)	92, 75	2.1 (1.6 to 2.6)
No method used	1030, 826	16.5 (15.5 to 17.6)	1173, 853	23.6 (22.3 to 25.0)
No vaginal sex in last year	463, 309	6.0 (5.4 to 6.7)	408, 271	7.4 (6.6 to 8.3)

*Denominator is Natsal population aged 16–44 years, excluding those who have never had vaginal intercourse.

†Numbers too small for patch (effective), cap/diaphragm in Natsal-3 (less effective), female condom (less effective), spermicides (less effective)

**Figure 1:**
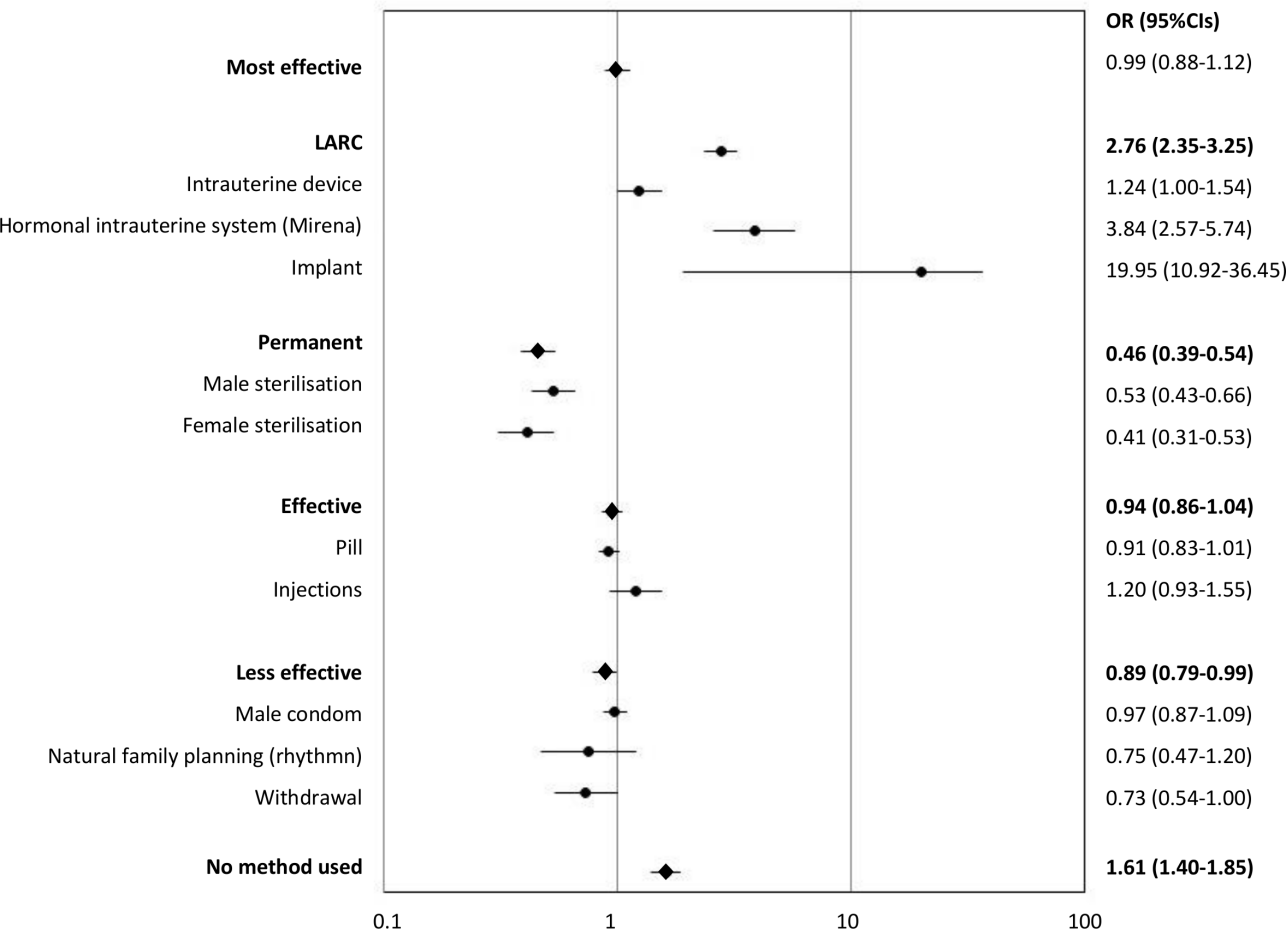
Changes between Natsal-2 and Natsal-3 in the odds of usual contraceptive method use in the last year: sexually experienced*Reporting having ever had vaginal intercourse among women aged 16–44 years. CI, confidence interval; OR, odds ratio.

No significant difference was observed in the odds of use of the most effective and effective methods between the surveys (figure 1). However, in the most effective category the overall change in likelihood of use masks considerable differences in contraceptive methods used within this category, particularly the move from permanent to reversible methods. Increases observed in odds of reporting current use of implants (OR 19.95, 95% CI 10.92 to 36.45) and the IUS (OR 3.84, 95% CI 2.57 to 5.74) were offset by a significant decline in odds of reporting use of male and female sterilisation (OR 0.53, 95% CI 0.43 to 0.66 and OR 0.41, 95% CI 0.31 to 0.53, respectively). Significant increases in the likelihood of reporting that they were currently not using any method were observed between Natsal-2 and Natsal-3 (OR 1.61, 95% CI 1.40 to 1.85).

The demographic and lifestyle characteristics by contraceptive method effectiveness category or non-method use among women who reported vaginal sexual intercourse in the last year for Natsal-3 are shown in table 2. Women who reported current use of female or male sterilisation were significantly more likely to be older (≥35 years), married and have two or more children. Women identifying as ‘white ’were significantly more likely to report use of sterilisation when compared with Asian women and women of mixed or other ethnicity. The only significant association found among LARC users was that they were more likely to have had two or more children.

**Table 2:** Prevalence of usual contraceptive method use in the last year by sociodemographic and lifestyle characteristics: Natsal-3

Characteristics Total	Denominator* (weighted/unweighted)	Most effective: sterilisation	Most effective: LARC	Effective	Least effective	No method used
		% (95% CI)	% (95% CI)	% (95% CI)	% (95% CI)	% (95% CI)
	4528 to 3184	8.9 (7.8 to 10.2)	14.3 (13.0 to 15.6)	35.3 (33.7 to 37.0)	23.9 (22.4 to 25.5)	17.5 (16.3 to 18.9)
**Sociodemographic**						
Age (years)						
16–17	244 to 118	0.0	14.5 (10.5 to 19.7)	47.5 (40.8 to 54.2)	33.2 (26.9 to 40.0)	4.9 (2.8 to 8.3)
18–19	353 to 185	0.0	12.3 (9.0 to 16.6)	56.0 (50.2 to 61.5)	25.8 (21.1 to 31.1)	5.9 (3.8 to 9.1)
20–24	928 to 544	0.3 (0.1 to 1.0)	13.7 (11.3 to 16.4)	50.4 (46.7 to 54.0)	24.1 (21.0 to 27.5)	11.5 (9.4 to 14.1)
25–29	1162 to 577	2.9 (2.1 to 4.0)	13.2 (11.0 to 15.7)	44.9 (41.7 to 48.1)	23.8 (20.8 to 27.1)	15.2 (13.0 to 17.7)
30–34	702 to 447	4.0 (2.7 to 6.0)	14.2 (11.5 to 17.4)	36.3 (32.6 to 40.1)	24.0 (20.6 to 27.7)	21.6 (18.5 to 25.0)
35–44	892 to 1074	18.5 (15.8 to 21.6)	15.4 (13.0 to 18.2)	20.8 (18.1 to 23.7)	22.6 (19.8 to 25.7)	22.7 (20.0 to 25.6)
Ethnicity						
White	3990 to 2772	9.9 (8.6 to 11.2)	14.4 (13.1 to 15.8)	37.3 (35.5 to 39.0)	22.3 (20.8 to 24.0)	16.1 (14.8 to 17.5)
Asian/Asian-British	214 to 181	2.9 (1.0 to 8.0)	8.7 (5.3 to 13.9)	12.0 (8.0 to 17.6)	41.2 (34.1 to 48.7)	35.2 (28.0 to 43.1)
Black/Black-British	122 to 94	4.1 (1.1 to 13.4)	18.9 (12.1 to 28.3)	21.6 (14.2 to 31.4)	32.5 (23.1 to 43.5)	23.0 (15.4 to 32.8)
Mixed/other	193 to 132	1.4 (0.4 to 4.8)	15.4 (9.9 to 23.3)	35.7 (28.2 to 44.0)	27.0 (20.2 to 35.1)	20.5 (14.8 to 27.5)
Religion						
None	2597 to 1720	8.7 (7.3 to 10.4)	14.4 (12.9 to 16.1)	39.2 (37.1 to 41.3)	22.0 (20.1 to 23.9)	15.7 (14.1 to 17.4)
Christian	1656 to 1253	10.2 (8.4 to 12.4)	14.5 (12.5 to 16.8)	33.2 (30.7 to 35.8)	24.3 (21.9 to 26.9)	17.6 (15.5 to 19.9)
Muslim	136 to 109	1.3 (0.2 to 8.5)	14.4 (8.8 to 22.8)	13.7 (8.2 to 22.0)	35.1 (26.9 to 44.4)	35.5 (27.1 to 45.0)
Hindu	55 to 38	0.0	6.3 (1.7 to 20.3)	10.9 (4.6 to 23.7)	52.3 (38.4 to 65.8)	30.5 (19.2 to 44.8)
Other	76 to 60	7.7% (3.1 to 18.1)	9.6% (3.8 to 22.3)	23.5 (15.5 to 34.0)	31.2 (20.6 to 44.2)	28.0 (17.6 to 41.3)
Academic qualification†						
No academic qualifications	369 to 241	9.9 (6.5 to 14.7)	16.0 (12.1 to 20.8)	25.7 (20.9 to 31.3)	17.6 (13.6 to 22.5)	30.8 (25.3 to 36.8)
Qualifications typically gained at age 16 years	1512 to 1077	15.2 (12.8 to 17.9)	15.4 (13.4 to 17.7)	33.6 (30.9 to 36.4)	18.0 (15.7 to 20.4)	17.8 (15.6 to 20.2)
Studying for/attained further academic qualifications	2415 to 1716	5.5 (4.3 to 6.9)	12.9 (11.3 to 14.7)	38.6 (36.5 to 40.7)	27.5 (25.4 to 29.7)	15.5 (13.9 to 17.3)
Index of Multiple Deprivation						
1–2 (Least deprived)	1566 to 1174	9.8 (8.0 to 12.0)	13.3 (11.4 to 15.4)	37.2 (34.5 to 39.9)	24.6 (22.2 to 27.2)	15.1 (13.1 to 17.4)
3	895 to 642	9.5 (7.1 to 12.5)	14.8 (12.2 to 17.8)	36.3 (32.9 to 40.0)	23.2 (20.1 to 26.7)	16.1 (13.5 to 19.2)
4–5 (Most deprived)	2068 to 1369	7.9 (6.4 to 9.8)	14.8 (13.0 to 16.9)	33.3 (30.9 to 35.8)	23.7 (21.5 to 26.1)	20.3 (18.2 to 22.5)
Relationship status						
Married	1408 to 1337	15.4 (13.3 to 17.9)	14.6 (12.5 to 16.9)	23.0 (20.8 to 25.5)	24.1 (21.6 to 26.8)	22.9 (20.5 to 25.4)
Cohabiting, not married	1023 to 727	6.3 (4.6 to 8.7)	14.7 (12.3 to 17.4)	41.9 (38.4 to 45.5)	21.6 (18.8 to 24.6)	15.5 (13.1 to 18.2)
Previously married	115 to 69	8.4 (3.7 to 18.0)	22.0 (14.6 to 31.9)	21.2 (14.7 to 29.6)	12.7 (7.9 to 19.8)	35.6 (25.5 to 47.2)
Never married	1972 to 1047	2.5 (1.6 to 3.8)	13.1 (11.4 to 14.9)	47.3 (44.7 to 49.8)	26.0 (23.7 to 28.5)	11.1 (9.6 to 12.7)
**Sexual and reproductive health**						
STI in last year						
No	2554 to 1750	8.2 (6.8 to 10.0)	12.1 (10.6 to 13.7)	35.6 (33.5 to 37.7)	25.6 (23.6 to 27.7)	18.5 (16.8 to 20.4)
Yes	100 to 55	3.7 (0.7 to 18.3)	9.0 (4.9 to 15.9)	53.1 (42.7 to 63.2)	22.7 (15.1 to 32.5)	11.5 (6.2 to 20.4)
Frequency of vaginal sex in last 4 weeks (n)						
0	791 to 530	5.1 (3.2 to 7.9)	12.6 (10.1 to 15.7)	31.7 (27.9 to 35.6)	25.3 (22.0 to 28.9)	25.3 (22.0 to 29.1)
1–4	1923 to 1415	9.3 (7.6 to 11.2)	13.1 (11.3 to 15.0)	33.0 (30.7 to 35.4)	27.2 (24.9 to 29.7)	17.4 (15.4 to 19.7)
5–9	1012 to 713	10.9 (8.4 to 13.9)	16.8 (14.3 to 19.7)	38.1 (34.8 to 41.4)	20.1 (17.4 to 23.1)	14.1 (11.7 to 17.0)
10+	772 to 504	9.2 (6.7 to 12.5)	15.6 (12.6 to 19.0)	42.3 (38.1 to 46.5)	19.2 (15.8 to 23.1)	13.8 (11.1 to 16.9)
Children (n)						
0	2075 to 1316	1.1 (0.7 to 1.8)	10.0 (8.6 to 11.7)	48.6 (46.2 to 51.1)	27.2 (24.9 to 29.5)	13.0 (11.4 to 14.9)
1	934 to 591	3.3 (2.0 to 5.4)	13.9 (11.3 to 16.9)	31.4 (28.1 to 34.9)	23.7 (20.7 to 27.0)	27.7 (24.4 to 31.3)
2+	1520 to 1277	19.6 (17.1 to 22.3)	18.7 (16.5 to 21.2)	23.4 (21.2 to 25.9)	20.8 (18.4 to 23.3)	17.5 (15.4 to 19.8)

*Denominator women <45 years who had vaginal sex in the last year, not pregnant.

†Ages >16 years.

LARC, long-acting reversible contraceptive; STI, sexually transmitted infection.

Use of effective methods peaked in the 18–19 years age group (56.0%, 95% CI 50.2 to 61.5). Asian women were significantly less likely than White women to report effective contraceptive method use, and usual use was significantly lower among women of Muslim or Hindu faith compared with Christians or those with no faith. Usual effective method use was significantly higher among women studying for or with academic qualifications compared with those with fewer qualifications. Prevalence was highest among unmarried women, including those cohabiting. Those with two or more children were significantly less likely to use these methods. Prevalence increased incrementally with frequency of sex and women using these methods were significantly more likely to report an STI in the last year.

Users of the less effective methods were significantly more likely to be younger (use peaking in the 16–17 years age group). There were differences in the ethnic and religious profiles of users. Around half of Asian women (41.2%, 95% CI 34.1 to 48.7) compared with 22.3% (95% CI 20.8 to 24.0) of White women reported current use of less effective methods. Higher prevalence of less effective use was reported among participants of Muslim and Hindu faiths. Use of less effective methods was significantly higher among those who were currently studying or had achieved higher academic qualifications, those having less frequent sex and those without any children.

Among those who reported vaginal sex in the last year and were not currently pregnant, 17.5% (95% CI 16.3-18.9) reported no current method, although this reduced to 9.0% (95% CI 7.9 to 10.3) when those who were trying to conceive or had had a hysterectomy were excluded from the analysis. The proportion of those who reported not currently using any contraceptive method increased incrementally with age, being reported by nearly a quarter of women aged 35–44 years (22.7%, 95% CI 20.0 to 25.6). No method use was highest among participants of Asian ethnicity and among those of Muslim and Hindu faith. Women without academic qualifications were significantly more likely to report no method use, and those living in more deprived areas were significantly more likely to report non-use when compared with women living in the least deprived areas. Non-use was also significantly higher among those who were currently or previously married, those who reported no vaginal sex in the last 4 weeks, and those with one child.

**Figure 2:**
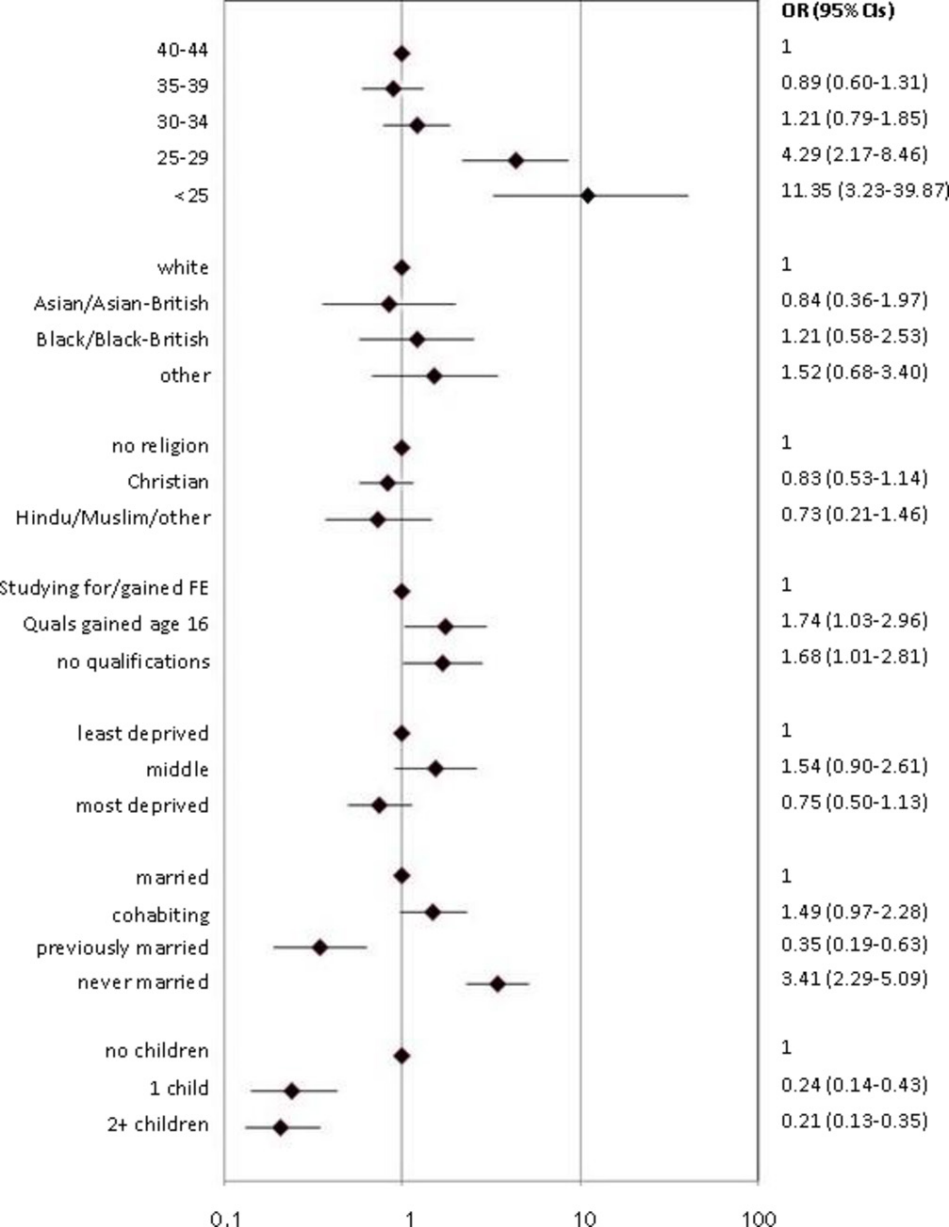
Changes between Natsal-2 and Natsal-3 in the odds of long-acting reversible contraceptive (LARC) method use in the last year by demographic and lifestyle characteristics: sexually active* women aged 16–44 years.*Reporting vaginal sex in the last year. CI, confidence interval; FE, further education; OR, odds ratio.

There was more than a 10-fold increase in under-25-year-olds reporting current use of a LARC method from Natsal-2 to Natsal-3 (OR 11.35, 95% CI 3.23 to 39.87) compared with women 40 years and above (figure 2). A four-fold increase over time was seen among the 25–29 years group. Use significantly increased among women who had never married compared with married women, (OR 3.41, 95% CI 2.29–5.09). A statistically significant increase was observed in the odds of use among those with fewer qualifications compared with those with more qualifications. A significant decline in the odds of current LARC use was observed among women with children compared with those with no children. No statistically significant differences were observed between the two surveys in relation to ethnicity, religion, and area of deprivation.

## Discussion

In both Natsal-2 and Natsal-3, one in five women reported that they were currently using a contraceptive method in the most effective category. The transition from less effective to most effective use overall corresponded with increased age, marriage, and having two or more children. No increase in overall most effective method use was observed among women, but significant increases in the odds of the implant and IUS use were seen between the two surveys. Increases in LARC use were particularly evident among the under-25s.

An increase in the use of LARC and a decline in sterilisation have been observed in other countries.^12 13^ In our analysis, while significant declines in male and female sterilisation were observed from 1999 to 2009, increased use of LARC was not sufficient to increase overall prevalence of most effective method use. Use of any contraceptive method is more cost effective than no method use; however, LARC methods have been found to be the most cost effective.^14 15^ A benefit of LARC methods over sterilisation is their reversibility, thus reducing both personal and financial costs for the small number of women who request reversal of sterilisation, and their greater suitability for younger people wanting highly effective protection against pregnancy. Lack of awareness and concerns about insertion and removal procedures, side effects and perceived ‘permanency’ of these methods are still barriers to uptake of LARC,^16–18^ but increasing accessibility and women’s knowledge (and removing any misconceptions) of these methods has been shown to have a significant impact on their uptake.^19^ Prescribing practice has also been found to be responsible for narrowing contraceptive choice.^20 21^ The large increase in most effective method use among the under-25s is promising and is an indication that knowledge and attitudes among both users and professionals may have shifted.

The oral contraceptive pill and condom are still the methods most commonly used, particularly among the young. Condoms are easily accessible and have the added benefit of STI protection. We grouped methods by the protection they offered against pregnancy rather than STIs. Condoms were grouped in the less effective category as we based the groups on typical failure rates, but if they are used consistently and correctly they offer good protection from both pregnancy and STIs. Nearly three-quarters of women report that they use condoms for exclusively pregnancy prevention compared with just over one in ten who use them exclusively for STI prevention.^22^ More effective contraceptive use could potentially have a negative consequence on STI rates if use of condoms declines as a consequence, so it is important that any education about these methods is given alongside messages on reducing risk of STIs. For the most part, it is still women who take responsibility for pregnancy prevention, and couples tend to move from condom to pill use as the relationship becomes more permanent and trusting.^23^

Contraceptive prevalence is high in Britain compared with other developed countries.^24^ However, another trend observed from our data was a significant increase between Natsal-2 and Natsal-3 in no method use among women who have had vaginal sex in the last year and were not currently pregnant. In their national probability survey of contraceptive behaviours among French women, Moreau and colleagues argue that even small changes in unmet contraceptive need contribute to the largest effects on unintended pregnancy rates within the general population.^25^ In Natsal-3, the highest prevalence of non-use (and less effective method use) among Asian participants and those of Muslim and Hindu faiths may be influenced by cultural norms around fertility, including less access to sexual and reproductive services, in some communities.^26 27^ It is notable that non-use was also higher among those without any academic qualifications and those living in more deprived areas, both factors which have been associated with unplanned pregnancy.^3^ While teenage pregnancies remain at a record low, abortion rates among women aged 25 years and older are rising and at a 10-year high.^28 29^

A strength of this study is that it provides national prevalence and trends at a population level. However, there are limitations that should be considered when interpreting the findings. The aim of our analysis was to present prevalence by contraceptive effectiveness category or non-use and to examine trends which would help inform public health initiatives and service provision. Therefore, we looked at crude, actual rates without adjustment for confounders to explore risk factors. Given the cross-sectional design of Natsal we could not reliability establish chronology and ascertain risk of unplanned pregnancy and current contraceptive method use or non-use. Numerous other socioeconomic and lifestyle trends including, perhaps most notably, the increase in the proportion of women entering higher education in Britain during the period in question, have had the potential to contribute to the increase in the use of LARC. Differences in population characteristics between Natsal-2 and Natsal-3 have been noted in other analyses, such as a decline in the proportion of women who are married or cohabiting.^30^ Although women were asked in Natsal about what sources they use to obtain contraceptive supplies they were not asked where they obtained specific methods, so it is not possible to determine who got what where and how this may have changed over time.^27^

Even a modest increase in effective contraceptive use results in financial savings and health gain.^31^ Eight in 10 women in the UK aged between 16 and 44 years report that they are currently using a method of contraception, demonstrating the need for accessible contraceptive services. The analysis in this article suggests some success in the first decade of the 21st century in widening access to LARC methods across the sociodemographic spectrum. However, since 2014 QOF payments to counsel pill and patch users about LARC methods have been scrapped and since 2015 community contraceptive services have faced major cuts to funding.^32^ Findings from Natsal-4 will be due from 2023 and will help us better understand the impact of changes in population demographics and lifestyles and service cuts on contraceptive use and reproductive outcomes.

## References

[1] Baird DT, Bajos N, Cleland J, et al Why after 50 years of effective contraception do we still have unintended pregnancy? A European perspective. Hum Reprod 2018;33:777–83. 10.1093/humrep/dey089 29659848

[2] Lakha F, Glasier A Unintended pregnancy and use of emergency contraception among a large cohort of women attending for antenatal care or abortion in Scotland. Lancet 2006;368:1782–7. 10.1016/S0140-6736(06)69737-7 17113427

[3] Wellings K, Jones KG, Mercer CH, et al The prevalence of unplanned pregnancy and associated factors in Britain: findings from the third National Survey of Sexual Attitudes and Lifestyles (Natsal-3). Lancet 2013;382:1807–16. 10.1016/S0140-6736(13)62071-1 24286786 PMC3898922

[4] National Institute for Health and Clinical Excellence (NICE) Long-acting reversible contraception (CG30), 2005 Available: https://www.nice.org.uk/guidance/CG30/chapter/1-Recommendations [Accessed 18 Jun 2019].

[5] Roland M Linking physicians' pay to the quality of care — a major experiment in the United Kingdom. N Engl J Med 2004;351:1448–54. 10.1056/NEJMhpr041294 15459308

[6] British Medical Association, NHS Employers Quality and outcomes framework guidance for GMS contract 2009/10. London, UK: BMA, NHS Employers, 2009 https://digital.nhs.uk/data-and-information/publications/statistical/quality-and-outcomes-framework-achievement-data/quality-and-outcomes-framework-2009-10

[7] Arrowsmith ME, Majeed A, Lee JT, et al Impact of pay for performance on prescribing of long-acting reversible contraception in primary care: an interrupted time series study. PLoS One;9:e92205 10.1371/journal.pone.0092205 PMC3973652 24694949

[8] National Statistics Statistics on sexual and reproductive health services (contraception). England 2017/18. Available: https://files.digital.nhs.uk/7B/862F70/srh-serv-eng-17-18-rep-rev.pdf [Accessed 17 Jun 2019].

[9] Erens B, Phelps A, Clifton S, et al Methodology of the third British National Survey of Sexual Attitudes and Lifestyles (Natsal-3). Sex Transm Infect 2014;90:84–9. 10.1136/sextrans-2013-051359 24277881 PMC3933071

[10] Sundaram A, Vaughan B, Kost K, et al Contraceptive failure in the United States: estimates from the 2006-2010 national survey of family growth. Perspect Sex Reprod Health 2017;49:7–16. 10.1363/psrh.12017 28245088 PMC5363251

[11] Cameron ST, Glasier A, Chen ZE, et al Effect of contraception provided at termination of pregnancy and incidence of subsequent termination of pregnancy. BJOG 2012;119:1074–80. 10.1111/j.1471-0528.2012.03407.x 22703553

[12] Darroch JE Trends in contraceptive use. Contraception 2013;87:259–63. 10.1016/j.contraception.2012.08.029 23040137

[13] Kavanaugh ML, Jerman J Contraceptive method use in the United States: trends and characteristics between 2008, 2012 and 2014. Contraception 2018;97:14–21. 10.1016/j.contraception.2017.10.003 29038071 PMC5959010

[14] Trussell J, Lalla AM, Doan QV, et al Cost effectiveness of contraceptives in the United States. Contraception 2009;79:5–14. 10.1016/j.contraception.2008.08.003 19041435 PMC3638200

[15] Mavranezouli I The cost-effectiveness of long-acting reversible contraceptive methods in the UK: analysis based on a decision-analytic model developed for a National Institute for Health and Clinical Excellence (NICE) clinical practice guideline. Hum Reprod 2008;23:1338–45. 10.1093/humrep/den091 18372257

[16] Baxter S, Blank L, Guillaume L, et al Views regarding the use of contraception amongst young people in the UK: a systematic review and thematic synthesis. Eur J Contracept Reprod Health Care 2011;16:149–60. 10.3109/13625187.2011.556762 21332384

[17] Glasier A, Scorer J, Bigrigg A Attitudes of women in Scotland to contraception: a qualitative study to explore the acceptability of long-acting methods. J Fam Plann Reprod Health Care 2008;34:213–7. 10.1783/147118908786000497 18854066

[18] Higgins JA Pregnancy ambivalence and long-acting reversible contraceptive (LARC) use among young adult women: a qualitative study. Perspect Sex Reprod Health 2017;49:149–56. 10.1363/psrh.12025 28419700 PMC5597464

[19] Secura GM, Allsworth JE, Madden T, et al The Contraceptive CHOICE Project: reducing barriers to long-acting reversible contraception. Am J Obstet Gynecol 2010;203:115.e1–115. 10.1016/j.ajog.2010.04.017 20541171 PMC2910826

[20] Cea Soriano L, Wallander M-A, Andersson S, et al Use of long-acting reversible contraceptives in the UK from 2004 to 2010: analysis using the health improvement network database. Eur J Contracept Reprod Health Care 2014;19:439–47. 10.3109/13625187.2014.948613 25139412

[21] Wellings K, Zhihong Z, Krentel A, et al Attitudes towards long-acting reversible methods of contraception in general practice in the UK. Contraception 2007;76:208–14. 10.1016/j.contraception.2007.05.085 17707718

[22] Cassell JA, Mercer CH, Imrie J, et al Who uses condoms with whom? Evidence from national probability sample surveys. Sex Transm Infect 2006;82:467–73. 10.1136/sti.2005.019117 17151032 PMC2563886

[23] Upadhyay UD, Raifman S, Raine-Bennett T Effects of relationship context on contraceptive use among young women. Contraception 2016;94:68–73. 10.1016/j.contraception.2016.02.025 26994674 PMC4884532

[24] Alkema L, Kantorova V, Menozzi C, et al National, regional, and global rates and trends in contraceptive prevalence and unmet need for family planning between 1990 and 2015: a systematic and comprehensive analysis. Lancet 2013;381:1642–52. 10.1016/S0140-6736(12)62204-1 23489750

[25] Moreau C, Bohet A, Trussell J, et al Estimates of unintended pregnancy rates over the last decade in France as a function of contraceptive behaviors. Contraception 2014;89:314–21. 10.1016/j.contraception.2013.11.004 24560475 PMC3972317

[26] Coleman DA, Dubuc S The fertility of ethnic minorities in the UK, 1960s–2006. Popul Stud 2010;64:19–41. 10.1080/00324720903391201 20087815

[27] French RS, Geary R, Jones K, et al Where do women and men in Britain obtain contraception? Findings from the third National Survey of Sexual Attitudes and Lifestyles (Natsal-3). BMJ Sex Reprod Health 2018;44:16–26. 10.1136/jfprhc-2017-101728 PMC6283328 29103003

[28] Department of Health and Social Care Abortion statistics, England and Wales, 2017 Available: https://assets.publishing.service.gov.uk/government/uploads/system/uploads/attachment_data/file/763174/2017-abortion-statistics-for-england-and-wales-revised.pdf29 [Accessed 17 Jun 2019].

[29] Information Services Division NHS Scotland Termination of pregnancy statistics. Year ending 31st December, 2018 Available: https://www.isdscotland.org/Health-Topics/Sexual-Health/Publications/2019-05-28/2019-05-28-Terminations-2018-Report.pdf [Accessed 17 Jun 2019].

[30] Mercer CH, Tanton C, Prah P, et al Changes in sexual attitudes and lifestyles in Britain through the life course and over time: findings from the National Surveys of Sexual Attitudes and Lifestyles (Natsal). Lancet 2013;382:1781–94. 10.1016/S0140-6736(13)62035-8 24286784 PMC3899021

[31] Sonnenberg FA, Burkman RT, Hagerty CG, et al Cost and cost-effectiveness of contraceptive methods. Contraception 2004;69:447–59. 15157789 10.1016/j.contraception.2004.03.008

[32] Advisory Group on Contraception. At tipping point. An audit of cuts to contraceptive services and their consequences for women. November,, 2018 Available: http://theagc.org.uk/wp-content/uploads/2018/11/At_tipping_point_AGC_Nov_18.pdf [Accessed 17 Jun 2019].

